# Morphological and trophic divergence of lake and stream minnows (*Phoxinus phoxinus*)

**DOI:** 10.1002/ece3.6543

**Published:** 2020-07-07

**Authors:** Kristin Scharnweber

**Affiliations:** ^1^ Department of Ecology and Genetics – Limnology Uppsala University Uppsala Sweden

**Keywords:** diet, freshwater fish, geometric morphometrics, natural selection, resource use, stomach content analyses

## Abstract

Phenotypic divergence in response to divergent natural selection between environments is a common phenomenon in species of freshwater fishes. Intraspecific differentiation is often pronounced between individuals inhabiting lakes versus stream habitats. The different hydrodynamic regimes in the contrasting habitats may promote a variation of body shape, but this could be intertwined with morphological adaptations to a specific foraging mode.

Herein, I studied the divergence pattern of the European minnow (*Phoxinus phoxinus*), a common freshwater fish that has received little attention despite its large distribution. In many Scandinavian mountain lakes, European minnows are considered as being invasive and were found to pose threats to the native fish populations due to resource competition. Minnows were recently found to show phenotypic adaptations in lake versus stream habitats, but the question remained if this divergence pattern is related to differences in resource use. I therefore studied the patterns of minnow divergence in morphology (i.e., using geometric morphometrics) and trophic niches (i.e., using stomach content analyses) in the lake Ånnsjön and its tributaries to link the changes in body morphology to the feeding on specific resources. Lake minnows showed a strong reliance on benthic Cladocera and a more streamlined body shape with a more upward facing snout, whereas stream minnows fed on macroinvertebrates (larvae and adults) to a higher degree and had a deeper body with a snout that was pointed down. Correlations showed a significant relationship of the proportion of macroinvertebrates in the gut and morphological features present in the stream minnows. The results of this study highlight the habitat‐specific divergence pattern in morphology and resource use in this ubiquitous freshwater fish. Consequently, interspecific interactions of invasive minnows and the native fish population could differ in the respective food webs and resource competition could target different native fish species in the contrasting habitats.

## Graphical abstract

The results of this study highlight the habitat‐specific divergence pattern in morphology and resource use in a ubiquitous freshwater fish, which may promote contrasting interspecific interactions in the respective food webs.

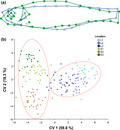

## 1 INTRODUCTION

Natural selection can evoke adaptive phenotypic divergence of populations (Endler, 1986; Rundle & Nosil, 2005; Schluter, 2000), which can lead to the formation of distinct populations or ecotypes (Svanbäck & Bolnick, 2005, 2007), and might even initiate speciation (Hendry, 2009). In freshwater fish species, underlying ecological factors responsible for diversifying patterns in populations may include different predation regimes (Scharnweber et al., 2013; Walsh & Reznick, 2009), parasite occurrence (Karvonen, Wagner, Selz, & Seehausen, 2018), or different hydrodynamic conditions (i.e., standing vs. flowing waters) (Ehlinger & Wilson, 1988; Webb, 1984). Lakes and the adjacent streams provide an ecological transition in space, that is, an ecotone, which can generate strong divergent selection, eventually promoting parapatric speciation (Berner, Grandchamp, & Hendry, 2009; Gavrilets, Li, & Vose, 2000; Schilthuizen, 2000). An environment that is characterized either by running or standing water may trigger divergence in fish populations with regard to morphological, physiological, developmental, or behavioral traits (Berner, Adams, Grandchamp, & Hendry, 2008; Walker, 1997). For example, to reduce the drag in the current, stream fishes often have a more streamlined body shape (Langerhans, 2008), which has been found, for example, in pumpkinseeds (*Lepomis gibbosus*) or rock bass (*Ambloplites rupestris*) (Brinsmead & Fox, 2002). However, divergence can also be based on resource use, often referred to as trophic polymorphism (Skúlason & Smith, 1995; Smith & Skúlason, 1996). Streams are generally characterized by a high abundance of macroinvertebrates (Demars, Kemp, Friberg, Usseglio‐Polatera, & Harper, 2012; Konrad, Brasher, & May, 2008), and a lower abundance of zooplankton (Chandler, 1937). Following the predictions of trophic polymorphism, morphological adaptations in fishes inhabiting lakes versus streams will have a contrasting result compared with predictions based on hydrodynamics. To forage within larger areas in the lake habitat may generate a more streamlined body, whereas swimming and maneuvering in the structurally complex stream habitat while searching for the more cryptic benthic prey will be supported by a deeper body (Anderson, 1984; Ehlinger, 1989; Robinson & Parsons, 2002). Such trophic polymorphism has been reported, for example, in lake and stream ecotypes of the three‐spined stickleback (*Gasterosteus aculeatus*) (Berner et al., 2008; Hendry, Taylor, & McPhail, 2002) and juvenile sockeye salmon (*Oncorhynchus nerka*) (Pavey, Nielsen, Mackas, Hamon, & Breden, 2010).

To understand the degree of variability in resource use, it is important to estimate ecologically significant diversity that occurs within a species (Bolnick et al., 2003). This has a particular relevance when the biological impact of an invasive species is estimated, that often comes from diet overlap and food competition with native species (Mooney & Cleland, 2001).

In this study, I investigated the patterns of divergence in the European minnow (*Phoxinus phoxinus*), a common freshwater fish that has received little attention despite its large distribution (Frost, 1943; Kottelat & Freyhof, 2007). It is an understudied fish species, despite its profound ecological impact when introduced to new areas, where it can become invasive and has the potential to modify original ecosystems (Museth, Borgstrøm, & Brittain, 2010; Museth, Hesthagen, Sandlund, Thorstad, & Ugedal, 2007; Næstad & Brittain, 2010). Minnows caught in lakes show a strong diet overlap with juvenile brown trout (Museth et al., 2010), and they are also regarded as one of the factors contributing to the reduced recruitment and growth of the native brown trout in lake habitats (Museth et al., 2007). Minnows were recently found to show phenotypic adaptations in lake versus stream habitats: Collin and Fumagalli (2011) studied minnow populations in Switzerland, and Ramler, Palandacic, Delmastro, Wanzenbock, and Ahnelt (2017) investigated minnows in Northern Italy and the Danube basin. The studies found opposing results: Collin and Fumagalli (2011) found stream minnows being more streamlined, a body form that is beneficial to reduce the drag in the current. In contrast, Ramler et al. (2017) reported that a streamlined body form was more pronounced in lake minnows compared to stream minnows and lake minnows also had larger heads compared to stream minnows. This might be due to habitat‐induced changes in head structures linked to different modes of foraging, as it is known, for example, from European perch (*Perca fluviatilis*) (e.g., Scharnweber, Strandberg, Marklund, & Eklöv, 2016; Svanbäck & Eklöv, 2002). However, evidence on trophic niche divergence, incorporating morphological adaptations in minnows inhabiting lake versus stream habitats is missing. Such information is crucial to evaluate interspecific competition between invasive minnows and native organisms.

Herein, I have analyzed stomach contents to understand the trophic niches during summer in minnows in the lake Ånnsjön, Central Sweden and its tributaries. This method has the advantage to provide a direct insight into the foraging ecology, giving information on ingested prey with a high taxonomic resolution (Hyslop, 1980; Manko, 2016). By combining the resource use assessment with morphological analyses by geometric morphometrics, I aimed to link the changes in body morphology to the individual resource use in the respective habitats. I predict that in stream minnows, the dietary contribution of macroinvertebrates would be higher compared with lake minnows. Furthermore, I predict that there is a relationship between morphology and dietary preference, indicating a specific body form when consuming specific prey.

## 2 MATERIAL AND METHODS

### 2.1 Sampling and study area

**FIGURE 1:**
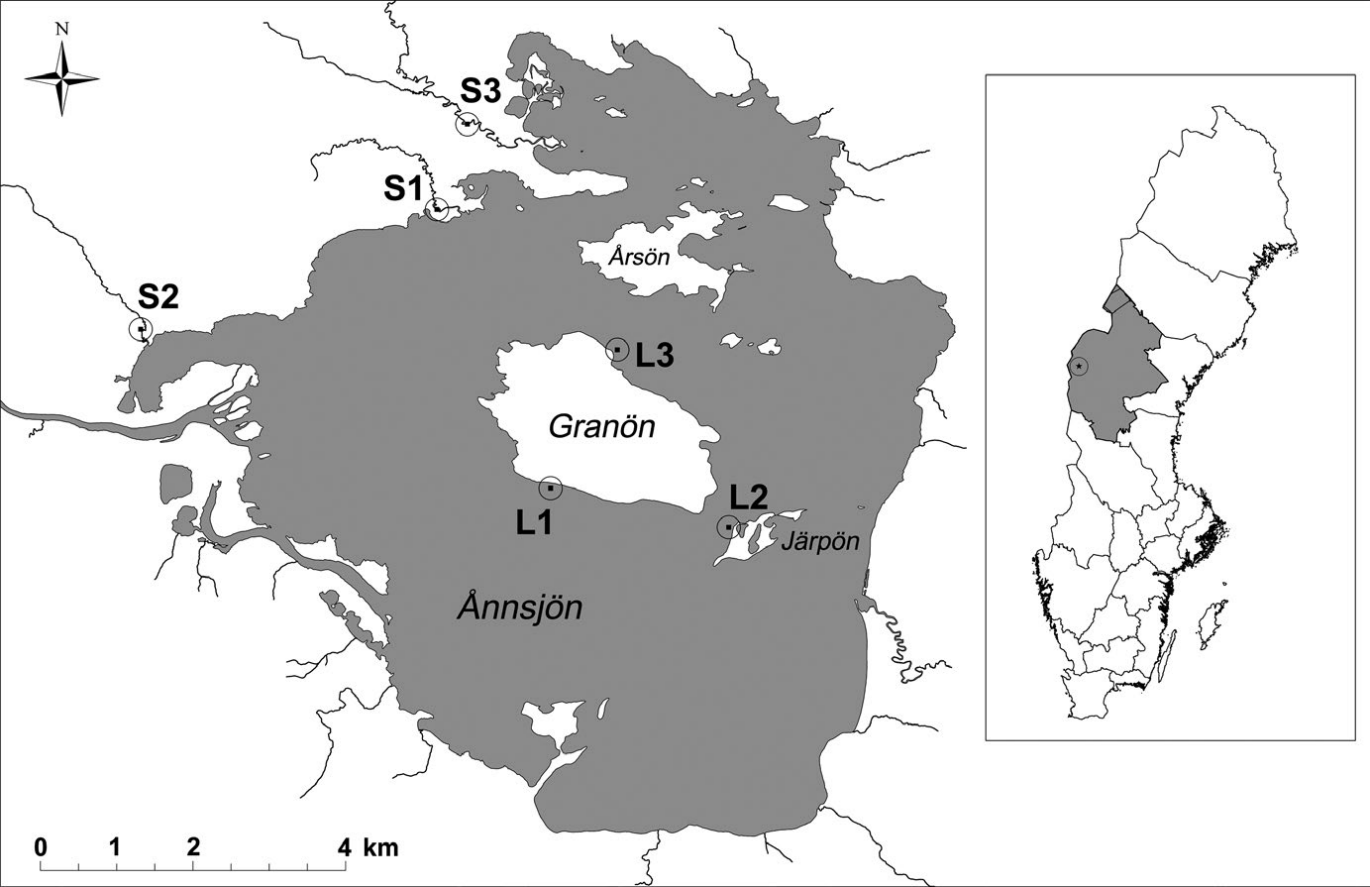
Map of the lake Ånnsjön and its position in Sweden including the locations where minnows were caught in the lake (L1, L2, L3) and the surrounding tributaries (S1, S2, S3). © Landmäteriet

The lake Ånnsjön is located in Central Sweden (63.261212°N, 12.567719°E) at an elevation of 526 m (Figure 1). The area of the lake comprises 57 km^2^ and most of it is relatively shallow (below 2 m deep), but the deepest point is 39.5 m (Bergwall & Berglund, 2010). Minnows are the most common fish species and the species‐poor fish community is further composed of brown trout *Salmo trutta*, Arctic charr *Salvelinus alpinus*, lake trout *Salvelinus namaycush*, and brook charr *Salvelinus fontinalis*. In August 2018, minnows were caught from three lake locations (L1, L2, L3; Figure 1) using gill nets (1 × 10 m with 6 mm mesh size), which were exposed for up to 12 hr. Furthermore, minnows were collected from three different slow‐flowing tributaries that were less than two km away from the lake: downstream Stor Klockbäcken (location S1), downstream Sjöviksbäcken (location S2), and downstream Kvarnbäcken (location S3) (Figure 1). In the streams, minnows were caught using an electrofishing approach and killed with an overdose of benzocaine. Fish were frozen to −20°C and transported to the laboratory at Uppsala University.

In total, 279 minnows were analyzed, 158 from the lake locations (L1: 52, L2: 52, L3: 54), and 121 in the streams (S1: 50, S2: 50, S3:21). In the laboratory, fish were thawed and subsequently individual length (to the nearest mm) was taken. For geometric morphometric analyses, a photograph was taken on the left side of the fish with fins stretched out. After taking the photograph, the entire gut was collected and kept frozen at −20°C for subsequent gut content analyses.

### 2.2 Geometric morphometrics

**FIGURE 2:**
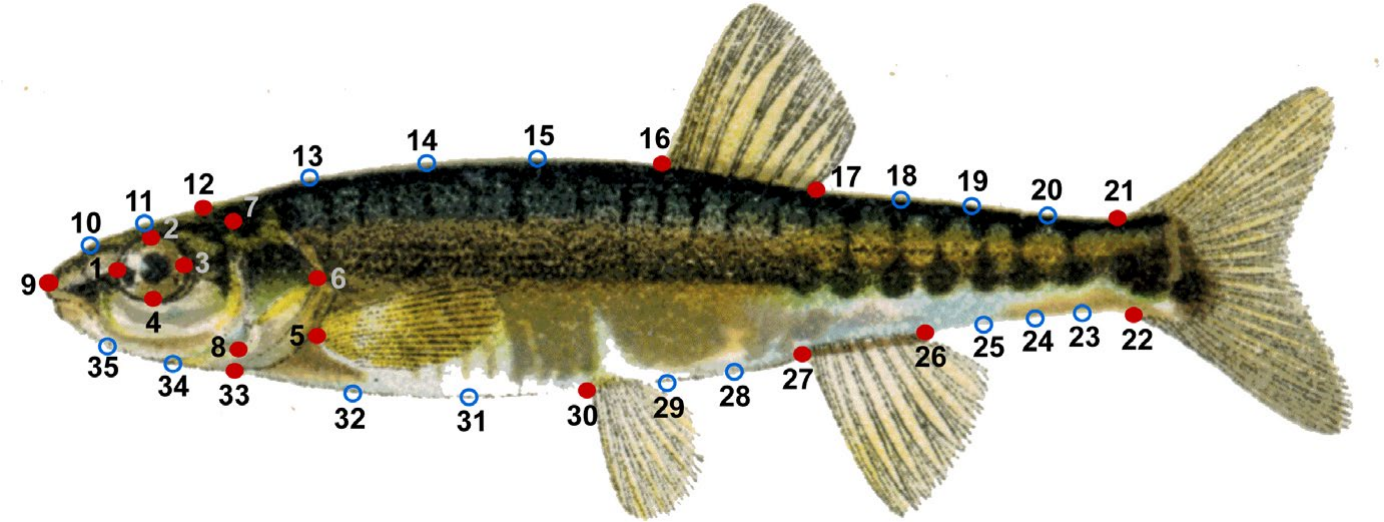
Position of the 35 digitized landmarks used in geometric morphometrics. Homologous landmarks (red dots): 1–4: most posterior, dorsal, anterior, and ventral point of orbit; 5: insertion of pectoral fin; 6–8: most posterior, dorsal, and ventral margin of operculum; 9: tip of the snout; 12: dorsal transition head to body; 16: anterior insertion of dorsal fin; 17: posterior insertion of dorsal fin; 21: dorsal insertion of caudal fin; 22: ventral insertion of caudal fin; 26: posterior insertion of anal fin, 27: anterior insertion of anal fin; 30: anterior insertion of pelvic fin; 33: ventral transition head to body. Semi landmarks (blue dots) were based on equidistant distances between homologous structures

The body morphology of individual minnows caught in lake and stream locations was analyzed using a landmark‐based geometric morphometric method (Bookstein, 1991). Digital lateral photographs were transferred to TPSdig2 (https://life.bio.sunysb.edu/morph/) and 35 landmarks were determined, including 18 homologous and 17 semi‐landmarks based on equidistant distances between structures (Mitteroecker & Gunz, 2009) (Figure 2).

### 2.3 Gut content analyses

Gut content was quantified from the entire gut of the minnows using a dissecting microscope. Gut fullness was estimated (five categories: 0, 0.25, 0.5, 0.75, 1) and used to weight the estimated volume proportion (equivalent to area proportion at uniform width) of each prey category observed in the sample which was estimated to the nearest 5%. Food items were classified as (a) benthic Cladocera [chydorid Cladocera (*Eurycercus* spp., *Alona* spp.,and *Chydorus* spp.)], (b) pelagic zooplankton [*Bosmina* spp., *Daphnia* spp., *Ceriodaphnia* spp., *Leptodora* spp., and Ostracoda], (c) macroinvertebrates [Amphipoda, Chironomidae, Ephemeroptera, Nematoda, Trichoptera, Bivalvia, Gastropoda, Coleoptera, Odonata, Arachnida, Oligochaeta, Polychaeta] and (d) terrestrial insects [Diptera imagoes (i.e. adults of aquatic Diptera larva)], and (e) unidentified items and mucus.

### 2.4 Statistical analyses

For minnow sampling, two different kind of gear was used (i.e., gillnets with one mesh size in the lake locations and electrofishing in the stream locations). Because of possible size differences in samples of lacustrine and riverine populations as a consequence of size‐selective sampling using gillnets in the lakes (Rudstam, Magnuson, & Tonn, 1984), an ANOVA with total length as dependent variable and location nested within habitat as independent variable was conducted. The assumptions of normal distribution and homogeneities of variances were met for the data used in this analysis.

Variation in morphology between the habitats (i.e., lake and stream) and locations was examined using MorphoJ v.1.06d (Klingenberg, 2011). No outliers were found in the morphological dataset when using the “Find outliers” function. To correct the shape data for body size, I used a regression of the shape scores (Procrustes coordinates) on size (centroid size) for each location separately and the residuals of this regression were used for all further analyses (Klingenberg, 2016). A discriminant function analysis (DFA) and a canonical variate analysis (CVA) were used to assess significance of shape differences between habitats. A second CVA was conducted for pairwise comparison between the six locations. The shape analysis was restricted to a maximum of 30 individuals of each location.

As minnows crush their food using pharyngeal teeth, many individuals solely had unidentified items and mucus in their guts (37.5% of all minnows caught) and these individuals were excluded from the analyses. Ordination of multivariate diet composition was based on Bray‐Curtis similarities and analyzed using a PERMANOVA with location nested within habitat, setting location as a random factor and habitat as a fixed factor. The significance of the model was tested with unrestricted permutations (999 permutations) with type III sums of squares. To test whether the contribution of the four diet categories (i.e., benthic Cladocera, pelagic zooplankton, macroinvertebrates, and terrestrial insects) differed between the individuals caught in the lake versus streams, I applied nonparametric Mann–Whitney *U* tests. I further conducted nonparametric Kruskal–Wallis tests with Bonferroni—adjusted Dunn's pairwise comparisons to analyze if the contribution of the four diet categories differed between the six different locations.

To test the relationship between resource use and minnow body shape, I used nonparametric Spearman's rank correlation on the individual proportions of macroinvertebrates in gut content and the first axis of the CVA (CV 1) from minnows caught in all locations.

PRIMER v 7.0.13 with the PERMANOVA add‐on (Primer E Ltd.) was used to analyze the multivariate dataset, whereas univariate analyses were conducted using IBM SPSS v.25 (IBM Corp.).

### 2.5 Ethical statement

The study was approved by the Umeå Animal Ethic Committee with permit number: A21‐2018. The permit for conducting electrofishing was received from the County Administrative Board Länsstyrelsen Jämtlands län.

## 3 RESULTS

### 3.1 Total length of minnows caught in the different locations

Minnows caught in the six different locations varied in total length between 4.9 and 8.2 cm. ANOVA revealed no significant difference between minnow length of the different locations nested within habitats.

### 3.2 Geometric morphometrics

**FIGURE 3:**
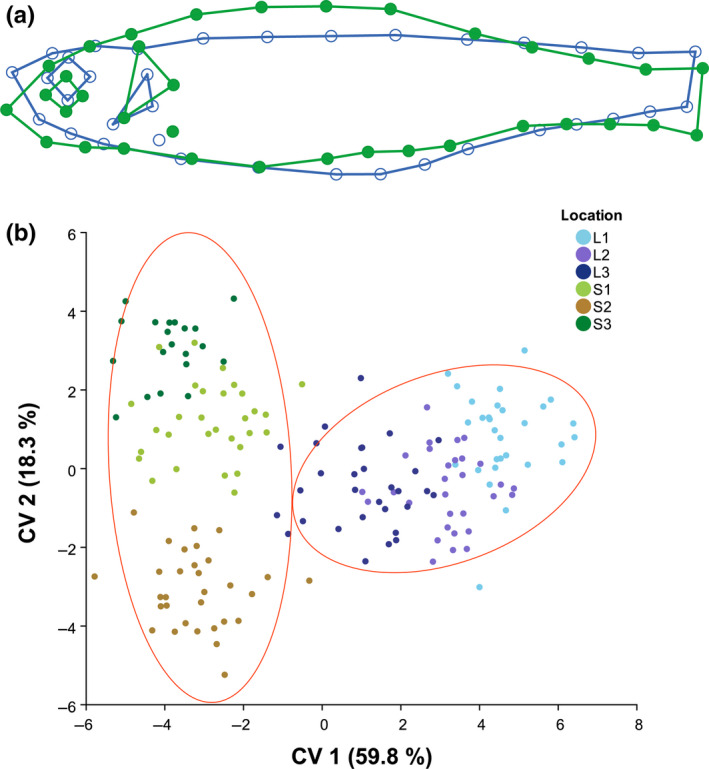
Results of geometric morphometrics. (a) Shape differences between minnows caught in the lake (blue line) and the streams (green line). Shape‐change outlines of Discriminant Function Analyses are magnified threefold. (b) Ordination of shape based on Canonical Variate Analyses of minnows caught in the six different locations with confidence ellipses (probability 0.9) drawn for lake and stream habitats, respectively

As revealed from DFA, differences in body morphology between minnow caught in the lake versus streams were significant (Mahalonobis distance *D* = 5.3026, *p* < .001). Further, DFA classified 90.0% of all lake individuals and 92.3% of all stream individuals correctly into the respective group. In general, lake minnows were characterized by an upward facing snout and body shape was more streamlined, whereas stream minnows showed a snout that was more pointed downward and the body was bulkier with a larger head (Figure 3a). Furthermore, stream minnows showed larger operculums, and a longer caudal peduncle (Figure 3a). The first axis of CVA (CV 1) explained 59.8% of the variation in the morphospace and along this axis, separation between minnows caught in lake and stream habitats occurred (Figure 3b). CV 2, which explained 18.3% of the variation, indicated variability in body shape between the minnows caught in the different streams (Figure 3b). Pairwise comparisons of minnow body shape between the locations showed significant differences between lake versus streams, but further also between L1 and L3 in the lake habitat (Table 1). As seen from the ordination of CVA, minnow morphology of individuals caught in L3 was most similar to stream minnows (Figure 3b).

**TABLE 1:** Results of Canonical Variate Analyses on pairwise comparison of body shape of minnows caught in the six different locations

	Lake locations						Stream locations					
	L1		L2		L3		S1		S2		S3	
	*S*	*p*	*S*	*p*	*S*	*p*	*S*	*p*	*S*	*p*	*S*	*p*
Lake locations												
L1												
L2	0.012	.118										
L3	**0.018**	**.003**	0.011	.220								
Stream locations												
S1	**0.037**	**<.001**	**0.031**	**<.001**	**0.027**	**<.001**						
S2	**0.041**	**<.001**	**0.034**	**<.001**	**0.028**	**.003**	0.017	.073				
S3	**0.035**	**<.001**	**0.029**	**.001**	**0.026**	**.002**	0.013	.253	0.019	.127		

Note

Depicted are Procrustes distances (*S*) among groups and the *p*‐value. Bold font depicts significant differences.

### 3.3 Gut content analyses

**FIGURE 4:**
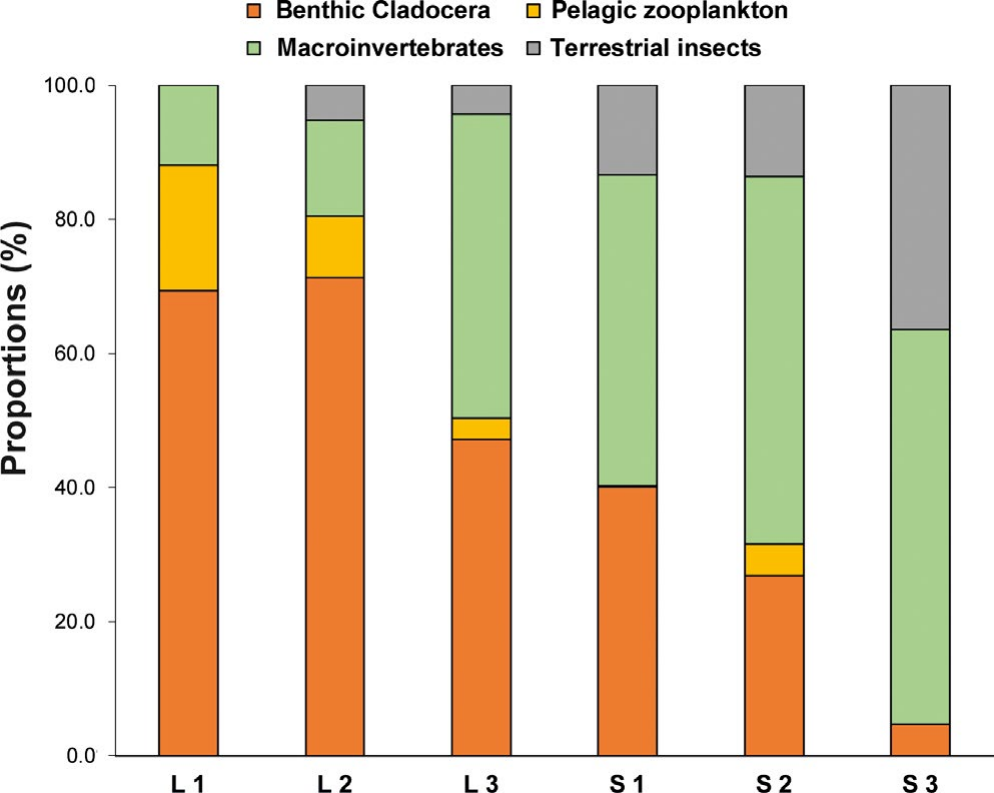
Average proportions of gut content of minnows caught in the lake (L1, L2, L3) and the streams (S1, S2, S3)

Gut content differed significantly between lake and streams (PERMANOVA: Pseudo‐*F*_5,278 _: 3.7748, *p* = .039), but further differed significantly between locations nested within habitats (PERMANOVA: Pseudo‐*F*_5,278 _: 6.8904, *p* = .001). The contribution of benthic Cladocera was significantly higher in lake locations (average 64.3% ± 45.5 *SD*) compared with streams (average 28.1% ± 41.0 *SD*) (Mann–Whitney *U*: *Z*_1_ = −3.807, *p* < .001, Table 2, Figure 4), whereas no significant difference could be found in the contribution of pelagic zooplankton between minnows caught in the lake (average 11.1% ± 31.2 *SD*) and streams (average 1.8% ± 11.3 *SD*). In contrast, the contribution of macroinvertebrates was significantly higher in streams (average 51.9% ± 46.3 *SD*) compared with lake locations (average 21.5% ± 38.8 *SD*) (Mann–Whitney *U*: *Z*_1_ = −5.600, *p* < .001, Table 2, Figure 4). Furthermore, the contribution of terrestrial insects was significantly higher in stream locations (average 18.2% ± 35.8 *SD*) compared with lake locations (3.1% ± 16.6 *SD*) (Mann–Whitney *U*: *Z*_1_ = −3.978, *p* < .001, Table 2, Figure 4). Between the locations, pairwise comparisons of the significantly different diet categories reflected the overall differences between lake and streams (Table 3). In addition, it showed variation in resource use between locations of the same habitat: minnows caught at location L3 had significantly lower proportions of benthic Cladocera in their guts compared with L2 (Table 3a, Figure 4). At this location, minnows were characterized by a higher contribution of macroinvertebrates, and no significant difference was found between this location and the stream locations S2 and S3, respectively (Table 3b, Figure 4). Furthermore, the proportion of terrestrial insects was significantly higher in location S3 compared with all other locations (Table 3c, Figure 4).

**TABLE 2:** Diet composition of minnows caught in the lake and streams

	Lake locations						Stream locations					
	L1 (*N* = 32)		L2 (*N* = 33)		L3 (*N* = 23)		S1 (*N* = 38)		S2 (*N* = 31)		S3 (*N* = 18)	
	Mean	*SD*	Mean	*SD*	Mean	*SD*	Mean	*SD*	Mean	*SD*	Mean	*SD*
*Eurycercus* spp.	66.3	47.0	71.1	41.1	42.8	48.1	29.1	42.1	8.9	27.4	1.9	3.9
*Alona* spp.	3.2	17.7	0.3	1.7	4.3	20.9	11.1	31.1	17.6	34.0	2.6	5.7
*Chydorus* spp.	0	0	0	0	0	0	0	0	0.3	1.8	0.1	0.5
*Bosmina* spp.	0	0	0	0	3.3	15.6	0.1	0.8	0	0	0	0
*Daphnia* spp.	12.5	33.6	3.0	17.4	0	0	0	0	0	0	0	0
*Ceriodapnia* spp.	3.1	17.5	6.1	24.2	0	0	0	0	0	0	0	0
*Leptodora* spp.	3.1	17.7	0	0	0	0	0	0	0	0	0	0
Ostracoda	0		0	0	0	0	0	0	4.8	18.8	0	0
Gammarus	2.2	12.4	1.7	9.6	4.3	20.9	0	0	0	0	0	0
Chironomidae	3.1	17.7	3.8	12.3	8.3	21.7	1.4	6.2	43.1	44.8	9.8	27.9
Ephemeroptera	6.3	24.6	0	0	23.5	40.9	0	0	0	0	0	0
Nematoda	0.2	0.9	0	0	0	0	0	0	4.2	18.6	0	0
Trichoptera	0	0	3.2	17.4	0	0	31.0	41.2	1.0	5.4	41.5	46.3
Bivalvia	0	0	0	0	0	0	2.5	15.4	3.3	8.7	0.6	2.4
Gastropoda	0	0	0	0	0	0	8.3	25.2	3.2	17.8	2.2	7.3
Coleoptera	0	0	0	0	0	0	0	0	0	0	4.9	20.7
Odonata	0	0	0	0	0	0	2.6	16.2	0	0	0	0
Arachnida	0	0	0	0	0.4	2.1	0.5	3.2	0	0	0	0
Oligochaeta	0	0	0	0	8.7	28.8	0	0	0	0	0	0
Polychaeta	0.2	0.9	5.7	22.9	0	0	0	0	0	0	0	0
Diptera adult	0	0	5.2	20.9	4.3	20.9	13.4	32.7	13.6	32.9	36.4	42.6
Σ benthic Cladocera	69.4	45.7	71.4	41.2	47.1	48.6	40.2	45.5	26.8	41.5	4.7	7.5
Σ pelagic zooplankton	18.7	39.6	9.1	29.2	3.3	15.6	0.1	0.8	4.8	18.8	0	0
Σ macro‐invertebrates	11.9	31.4	14.4	30.4	45.3	49.3	46.4	46.3	54.7	48.0	58.9	44.6
Σ terrestrial insects	0	0	5.2	20.9	4.3	20.9	13.4	32.7	13.6	32.9	36.4	42.6

Note

Depicted are averages and standard deviation (*SD*) of the percentage of gut volume of each item, or sums of benthic Cladocera, pelagic zooplankton, macroinvertebrates and terrestrial insects, including the sample size (*N*).

**TABLE 3:** Results of Kruskal–Wallis tests on pairwise comparison of volumetric proportion of (a) benthic Cladocera, (b) macroinvertebrates, and (c) terrestrial insects in the gut of minnows caught in the six different locations

	Lake locations			Stream locations		
	L1	L2	L3	S1	S2	S3
(a) Benthic Cladocera: *H*_5_ = 28.171, *p* < .001						
Lake locations						
L1						
L2	1.000					
L3	0.243	**0.045**				
Stream locations						
S1	0.761	0.142	1.000			
S2	**0.036**	**0.004**	1.000	1.000		
S3	**0.007**	**0.001**	1.000	0.709	1.000	
(b) Macroinvertebrates: *H*_5_ = 38.102, *p* < .001						
Lake locations						
L1						
L2	1.000					
L3	0.577	1.000				
Stream locations						
S1	**0.004**	**0.042**	1.000			
S2	**<0.001**	**0.001**	0.480	1.000		
S3	**<0.001**	**0.003**	0.390	1.000	1.000	
(c) Terrestrial insects: *H*_5_ = 33.018, *p* < .001						
Lake locations						
L1						
L2	1.000					
L3	1.000	1.000				
Stream locations						
S1	0.724	1.000	1.000			
S2	0.884	1.000	1.000	1.000		
S3	**<0.001**	**<0.001**	**<0.001**	**0.002**	**0.004**	

Note

Depicted are the results of the overall test, and adjusted *p*‐value (Dunn‐Bonferroni correction) for pairwise comparisons of locations. Bold font depicts significant differences. No significant difference was found in the contribution of pelagic zooplankton between the locations, thus no pairwise comparisons are reported.

### 3.4 Relationship between resource use and morphological distance

**FIGURE 5:**
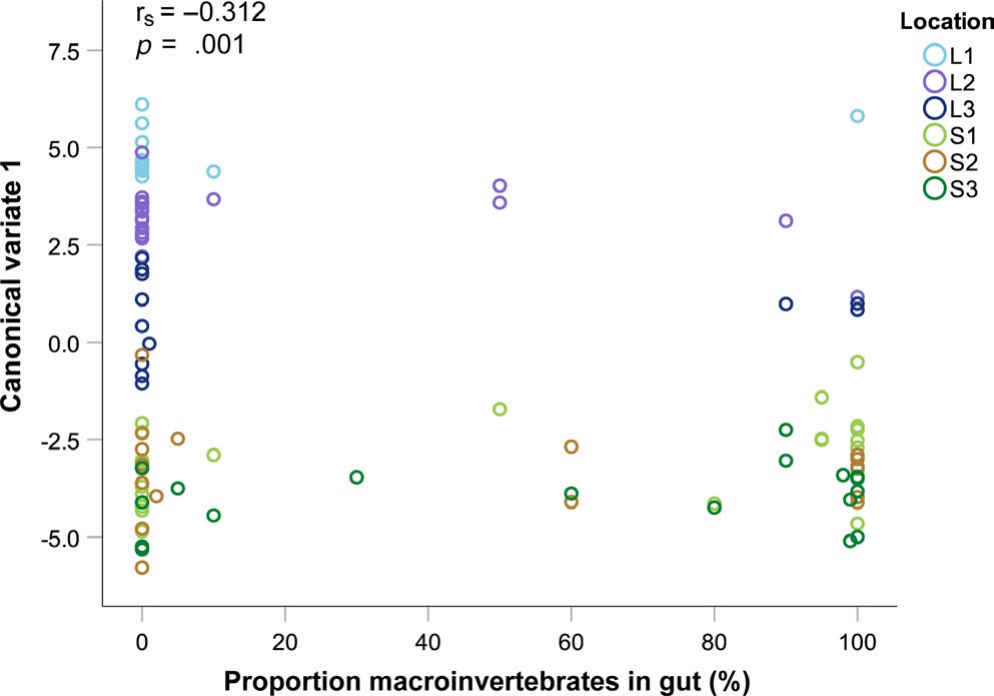
Relationship between proportion of macroinvertebrates in the minnow guts and morphology (canonical variate 1), including results of nonparametric Spearman's rank correlation

Along the first axis of CVA (CV 1), more negative CV‐values were associated with the stream‐body shape (Figure 3b). Spearman's rank correlation showed a significant negative relationship between the dietary contribution of macroinvertebrates and the morphological distances (i.e., values of CV 1) (*r*_s_ = −.312, *p* = .001, Figure 5).

## 4 DISCUSSION

Morphology differed between minnows caught in the lake and streams, with minnows caught in the lake being more streamlined with a mouth facing more upwards, and stream minnows having a deeper body and a mouth that was facing more downwards. This habitat‐specific body shape was associated with the use of a specific set of resources, indicating that minnow morphology is an adaptation to a specific foraging mode (Robinson & Wilson, 1994; Skúlason & Smith, 1995). Minnows caught in the lake Ånnsjön generally ingested more zooplankton, compared with minnows caught in the adjacent streams, which ingested macroinvertebrates and terrestrial insects to a higher degree. Thus, my results are in line with previous findings on minnow diet (Frost, 1943; Michel & Oberdorff, 1995). However, previous studies on minnow diet reported that feeding of lake minnows on pelagic zooplankton was pronounced (Frost, 1943; Michel & Oberdorff, 1995), whereas in the results presented herein, this proportion was minor and not significantly different to the proportions ingested by stream minnows. Instead, ingested zooplankton belonged to chydorid Cladocera, which do not live in the open‐water zone, but are instead benthic species that are plant‐ and bottom‐associated (Adamczuk, 2014; Goulden, 1971). Therefore, lake and stream minnows both fed on prey living in the same zone, but exhibit different morphological adaptations. To my knowledge, this is the first time that such a particular trophic divergence has been reported, but fine‐scale differences in trophic polymorphism are common (Hawley, Rosten, Christensen, & Lucas, 2016; Thomas et al., 2019). Morphological divergence although the prey lives in similar zones can be attributable to the fact that swimming activities related to feeding on the specific prey are very different in the two contrasting habitats. Streams provide structurally complex habitats and swimming requires maneuvering through vegetation to search for the rather large, but cryptic macroinvertebrates (Anderson, 1984; Ehlinger, 1989). In contrast, lake minnows need to swim more persistently to feed on the numerous, but small prey, which can be supported by a more streamlined body form (Robinson & Parsons, 2002; Webb, 1984). Furthermore, it has to be noted that in this study, only summer feeding was studied. While stomach content analyses have the strong advantage to obtain taxonomically highly resolved data, it provides only a snapshot of the recently ingested prey (Hyslop, 1980; Manko, 2016; Scharnweber et al., 2016). Potentially, proportion of ingested pelagic zooplankton might be seasonally variable and pelagic zooplankton species form a more essential part of minnow diet than suggested by this single sampling campaign.

In addition to differences in body depth and facing of the snout, stream minnows showed larger operculums. A larger operculum could simply be attributed to the fact that heads were larger in stream minnows. However, the operculum functions as a one‐way valve, regulating the inflow of water in the opercular cavity and by that, forms an integral part in the feeding apparatus necessary for suction feeding (Day, Higham, Holzman, & Van Wassenbergh, 2015). It can only be speculated if a larger operculum is connected to higher suction abilities that are needed to feed on the larger macroinvertebrates that form the dominant prey in stream minnows.

The major shape difference between minnows caught in lake and stream habitats was a snout that was facing more upwards or downwards, respectively. Such a finding resembles a common methodological artifact in geometric morphometric studies, termed the “arching effect,” which stems from imperfect positioning during photography, or from shrinking during long‐term storage (Valentin, Penin, Chanut, Sévigny, & Rohlfk, 2008). For the dataset presented herein, I could demonstrate a concise difference in body shape between minnows caught in the lake and streams evidenced by correct classification of DFA in >90.0% of all cases, whereas effects from arching would occur randomly across the individuals examined. I am therefore confident that my results have a biological implication and are not based on a methodological artifact.

Resource polymorphism will lead to intraspecific divergence within a single population (Skúlason & Smith, 1995; Smith & Skúlason, 1996) and can be seen as an early stage of speciation (Berner et al., 2009; Hendry, 2009). As predicted from niche evolution theory, morphological divergence will reduce competition as less prey items are shared (Bolnick et al., 2003; Dieckmann & Doebeli, 1999), and this pattern could also be demonstrated empirically in the perch‐roach system (Svanbäck, Eklöv, Fransson, & Holmgren, 2008). Intraspecific differentiation may initially emerge from phenotypic plasticity (Pfennig et al., 2010), and depending on the stability of the selective regime, divergent phenotypes may become genetically fixed (Crispo, 2008; Thibert‐Plante & Hendry, 2011). Unfortunately, genetic data for the minnows of this study are not available. Future studies to investigate the level of genetic differentiation are needed to characterize the position of the morphotypes of the European minnow in Sweden along the specification continuum that could vary from adaptive variation to complete reproductive isolation (Berner et al., 2009; Hendry, 2009; Nosil, Harmon, & Seehausen, 2009). However, results of morphological divergence, but also resource use showed a strong variation within the habitats, between the different locations. Individuals caught at location S3 were feeding to a greater extent on terrestrial insects, compared with individuals caught in the other two streams. Previous studies have linked the type of adjacent vegetation and canopy cover to the degree of surface prey ingested by stream fishes (Nakano & Murakami, 2001; Ryan & Kelly‐Quinn, 2015). The streams studied herein were not covered by canopy, but compared to the two other stream that were meandering through peat meadows of mosses and sedges without any higher vegetation, small willow shrubs were growing along the shores of S3. Furthermore, individuals caught at location L3 were feeding to a greater extent on macroinvertebrates and ingested fewer zooplankton than individuals caught at L1 and L2 and body shape was more similar to stream minnows. Such variation indicates a strong degree of plasticity in resource‐morph formation, which would suggest that differences between minnows of the two adjacent habitats might not be genetically fixed. In contrast to the other lake habitats, water plants were abundant at L3, which could provide suitable microhabitats for macroinvertebrates. To understand the driving forces behind the variation in the degree of minnow divergence, estimates of prey abundances at the different locations are needed.

In contrast to my results that are in accordance with the ones of Ramler et al. (2017), Collin and Fumagalli (2011) found minnows inhabiting stream habitats in Switzerland to be more streamlined compared to conspecifics living in lakes. However, besides attributing these morphological adaptations to the hydrodynamic conditions occurring in stream habitats, they further reported a high predation pressure present in the lake habitats. A deeper body shape can be seen as advantageous under such kind of ecological conditions, as muscle mass may enhance a rapid acceleration to escape predators (Langerhans, Layman, Langerhans, & Dewitt, 2003; Walker, Ghalambor, Griset, McKenney, & Reznick, 2005), and a deeper body can provide refuge from gape‐limited predators (Brönmark & Miner, 1992). Potentially, predation pressure could influence the strength and direction on the correlation of minnow morphology and diet, but further laboratory experiments are needed to resolve this relationship.

Scandinavian mountain lakes are characterized by a low species richness of fish. In many of these often remote lakes minnows were introduced by anglers as life bait (Museth et al., 2007). They are considered as being invasive, due to the fact that they can reach high densities, as for example, in the lake Ånssjön (Bergwall & Berglund, 2010). Næstad and Brittain (2010) further showed that they have the ability to modify lake food webs, thus being responsible for a zoobenthos assemblage with a dominance of Chironomidae and Oligochaeta, and a low abundance of *Gammarus lacustris*. As lake minnows show a strong diet overlap with juvenile brown trout (Museth et al., 2010), they are also regarded as one of the factors contributing to the reduced recruitment and growth of the native brown trout in lake habitats (Museth et al., 2007). My results presented herein indicate that minnows inhabiting stream habitats may rely on different resources than the individuals inhabiting lakes. Therefore, interspecific competition targets at different species in these contrasting habitats and patterns observed in lake habitats cannot be directly transferred to the interactions occurring in stream habitats. Nonetheless, the introduction of minnows into stream habitats may also pose a similar threat for the native fish populations of the stream, if a diet overlap would occur. Certainly, future studies need to determine the consequences of minnow invasions on the stream food webs.

## CONFLICT OF INTEREST

The author declares no conflict of interest.

## AUTHOR CONTRIBUTION

**Kristin Scharnweber:** Conceptualization (lead); data curation (lead); formal analysis (lead); funding acquisition (lead); investigation (lead); methodology (lead); project administration (lead).

## Data Availability

The data that support the findings of this study are openly available in DiVA at https://uu.diva‐portal.org, reference number: urn:nbn:se:uu:diva‐389472.
